# Next-generation sequencing at Merck-Boston

**DOI:** 10.1186/1753-6561-6-S6-P36

**Published:** 2012-10-01

**Authors:** Richard Stevens

**Affiliations:** 1 Affiliation: tbc

## 

Next-generation sequencing (NGS) is quickly replacing other methods for determining expression profiling of RNA as well as single-nucleotide variations within the genomes of both model organisms and human samples. At the Boston facility of Merck Research Laboratories, we concentrate on preparation and sequencing of samples where the research needs cannot be met by commercial venders. These unmet needs may be due to either availability of up-to-date protocols or to deadline constraints. In addition to validating and developing new library-construction and sequencing protocols, we also evaluate commercial vendors in consideration for future outsourcing. One of these current projects is an evaluation of transcriptome sequencing and profiling of formalin fixed paraffin embedded (FFPE) samples from Merck's current and past clinical studies. FFPE samples are readily available and easily stored but create difficulties in NGS analysis due to the low quality of the purified nucleic acids. Here, we compare techniques for transcriptome sequencing to microarray profiling of RNA purified from FFPE tissues as well as their mirrored fresh frozen counterparts.

